# Supplementary material for: Enhanced Malignancy Prediction of Small Lung Nodules in Different Populations Using Transfer Learning on Low-Dose Computed Tomography
Source: Diagnostics (Basel). 2025 Jun 8;15(12):1460. doi: 10.3390/diagnostics15121460 (PMC12192116; doi:10.3390/diagnostics15121460)
Supplement: Supplementary file 1 [file diagnostics-15-01460-s001.zip › diagnostics-3631834-supplementary.pdf]

Supplementary materials

Table S1. CT scanners and imaging parameters in the CGH and NLST datasets

CT scanners and imaging parameters in the CGH dataset						
Manufacturer Model Name	Patient Number	Pixel Spacing (mm)	Matrix Size	Slice Thickness (mm)	Tube Voltage (kVp)	Tube Current (mA)
Aquilion 640	3	0.567-0.728	512×512	3	120	60-103
Aquilion ONE	235	0.488-0.782	512×512	3-5	120	60-130
Aquilion Prime SP	74	0.518-0.782	512×512	3-5	120	20-429
Brilliance	54	0.483-0.684	512×512	1-5	120	48
Brilliance 64	203	0.488-0.781	512×512	3-5	120	40-246
Ingenuity CT	19	0.580-0.771	512×512	2-5	120	40-120
Sensation 16	37	0.602-0.912	512×512	2-5	120	60-384
Vereos PET/CT	3	0.547-0.645	512×512	3-5	120	73-106
CT scanners and imaging parameters in the NLST dataset						
Manufacturer Model Name	Patient Number	Pixel Spacing (mm)	Matrix Size	Slice Thickness (mm)	Tube Voltage (kVp)	Tube Current (mA)
Aquilion	20	0.543-0.781	512×512	2	120	80-160
HiSpeed QX/i	39	0.508-0.781	512×512	2.5	120	80
LightSpeed Plus	74	0.527-0.779	512×512	2.5	120-140	50-205
LightSpeed QX/i	167	0.527-0.957	512×512	2.5	120-140	40-100
LightSpeed16	35	0.508-0.764	512×512	2.5	120-140	50-130
Mx8000	48	0.533-0.781	512×512	3.2	120	60-187
Sensation 16	50	0.492-0.930	512×512	2-5	120	72-140
Volume Zoom	166	0.488-0.977	512×512	2-5	120-140	80-320
Unknown	1	0.676	512×512	2.5	120	120

Table S2. The malignancy of three SLN types in the CGH and NLST dataset.

	CGH dataset	NLST dataset	p values
**Solid**			0.334
Benignness	181 (66.54%)	299 (70.02%)	
Malignancy	91 (33.46%)	128 (29.98%)	
**Partial solid**			0.005**
Benignness	105 (50.97%)	43 (71.67%)	
Malignancy	101 (49.03%)	17 (28.33%)	
**GGO**			< 0.001***
Benignness	68 (35.60%)	85 (75.22%)	
Malignancy	123 (64.40%)	28 (24.78%)	

** $p<0.01$, *** $p<0.001$

Table S3. The network architecture for the DL models.

	**ResNet-18**	**U-Net**	**2C-U-Net**
Layer number	71	49	67
Node per layer	[64, 128, 128, 256, 256, 512]	[16,32,64,32,16]	[16,32,64,32,16]
Normalization layer	Batch normalization	Batch normalization	Batch normalization
Activation layer	Leaky ReLU	Leaky ReLU	Leaky ReLU
Block number	3	3	3
Loss function	Cross entropy	Cross entropy	Cross entropy

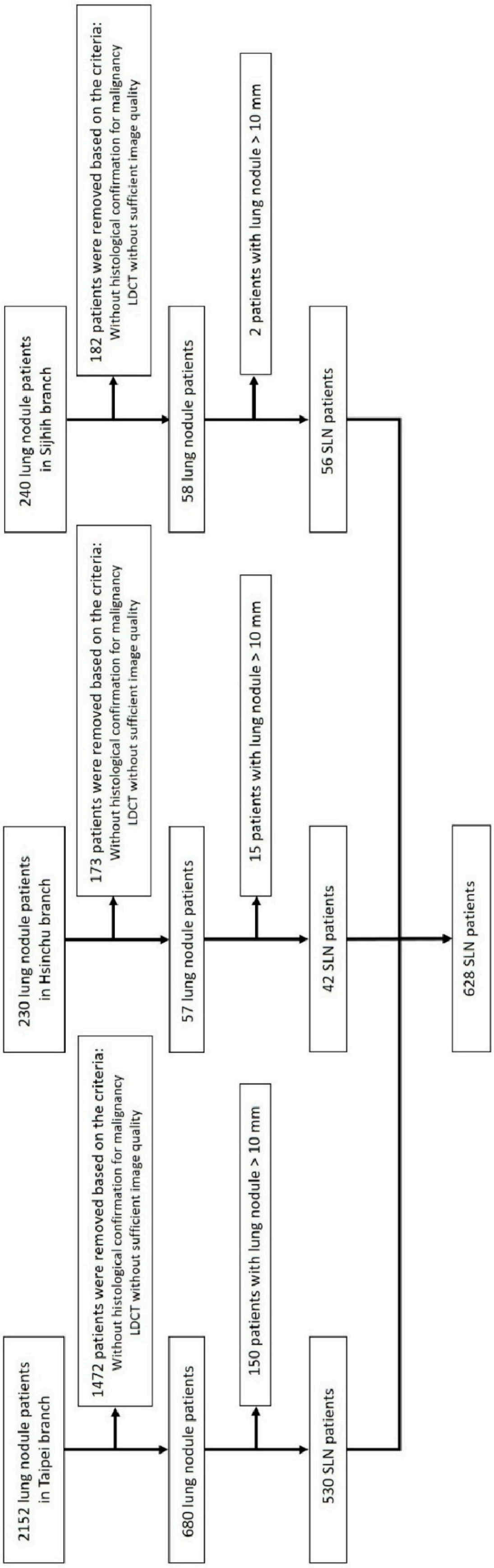

Figure S1. The patient enrollment flowchart for the CGH dataset.

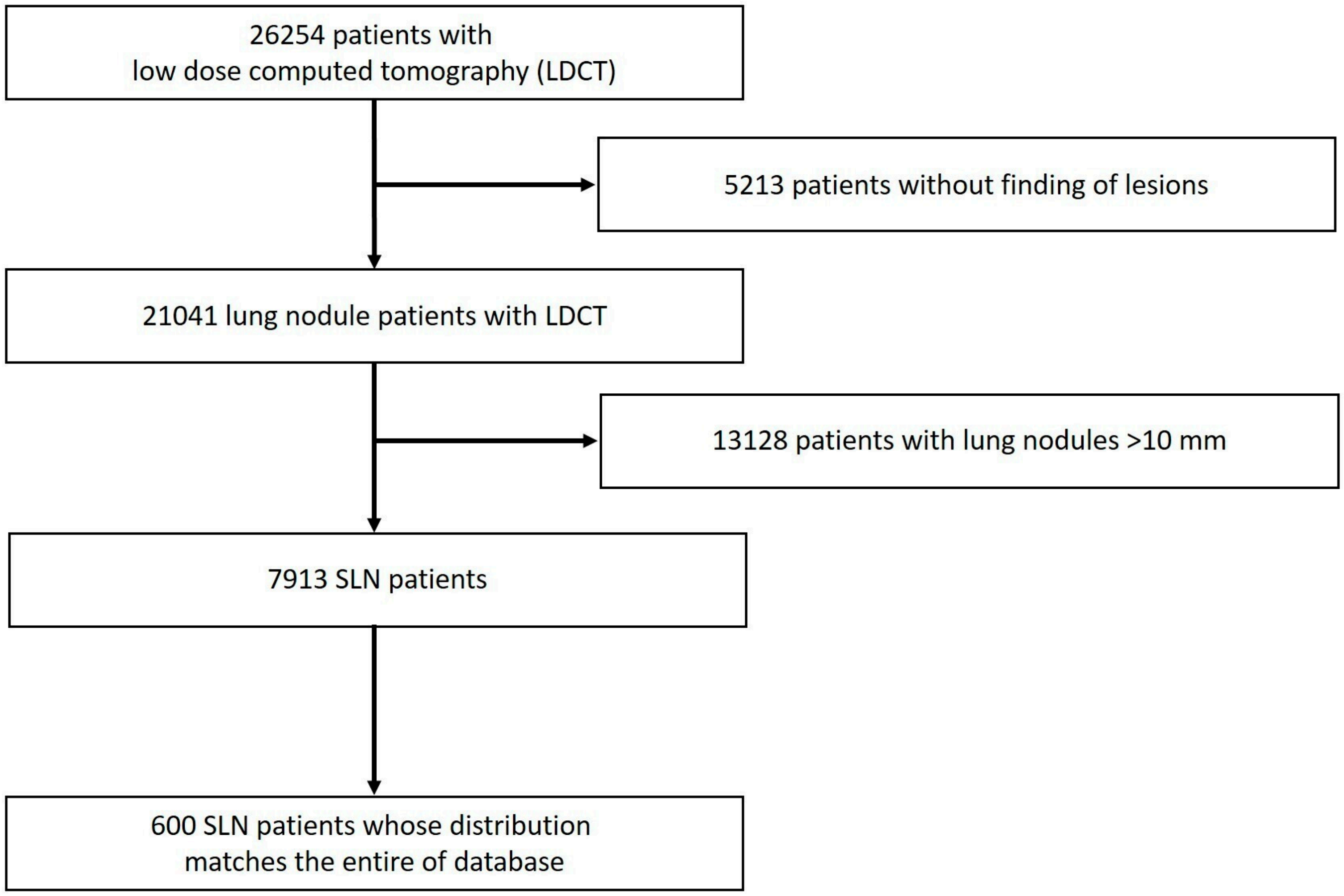

Figure S2. The patient enrollment flowchart for the NLST dataset.

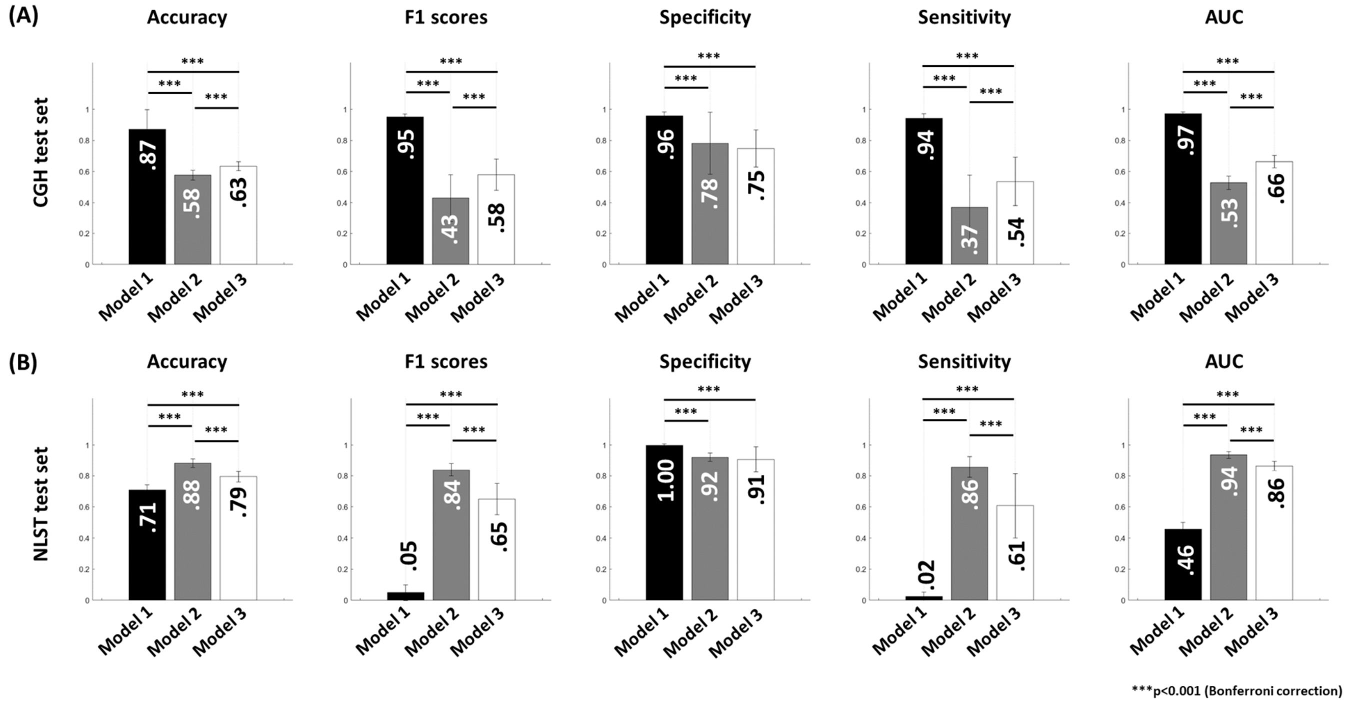

Figure S3. Performance comparisons in the target dataset among specialized models and pooling model. The accuracy, F1 scores, specificity, sensitivity, and AUC of **Model 1 (CGH model)**, **Model 2 (NLST model)**, and **Model 3 (Pooling model)** in the test set of (A) the CGH dataset and (B) the NLST dataset, respectively. **p<0.01, ***p<0.001

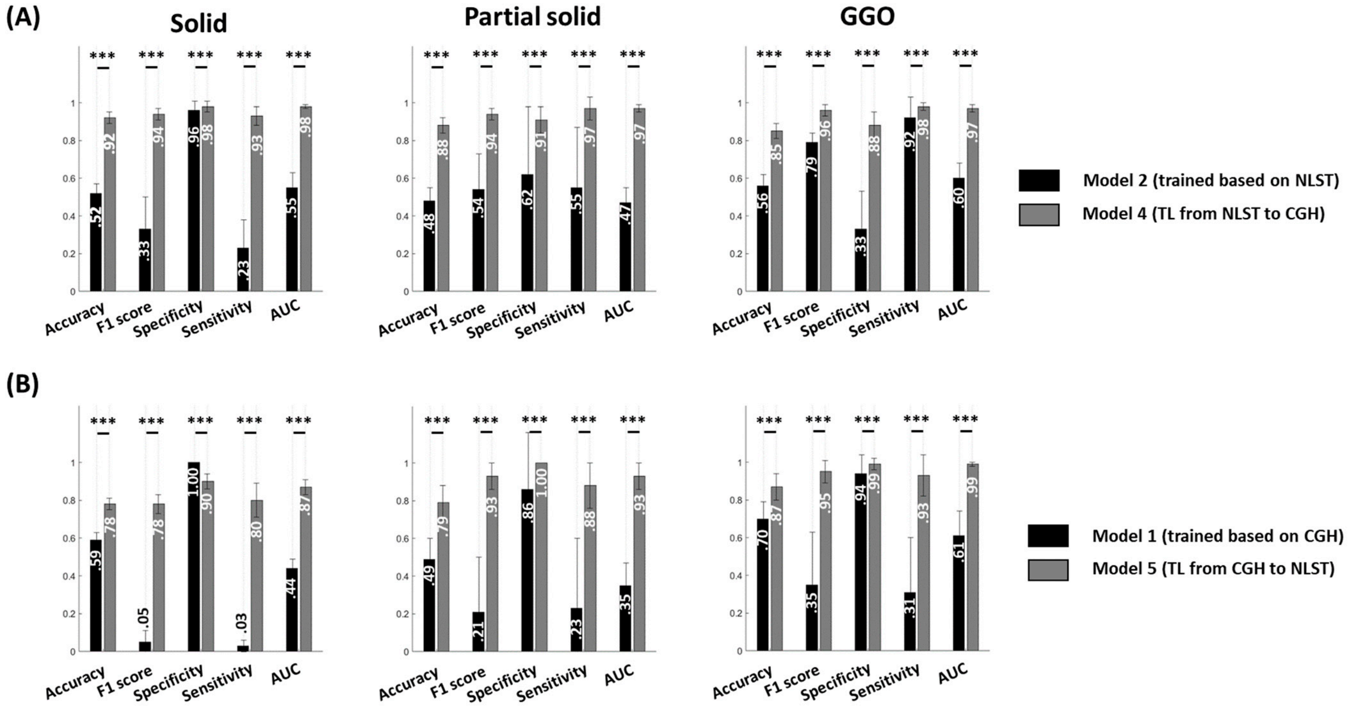

Figure S4. Comparisons of accuracy, F1 score, specificity, sensitivity, and AUC between models with and without TL in the target datasets in different SLN types. (A) Comparison between **Model 2** and **Model 4** in the test set of the CGH dataset, (B) Comparison between **Model 1** and **Model 5** in the test set of the NLST dataset. Black and white bars represent the performance of specialized and TL models, respectively. ***$p<0.001$

We conducted comparisons between the applied two-channel U-Net (2C U-Net) [1] and other two methods, including ResNet-18 [2] and typical U-Net [3], for predicting SLN malignancy. All three models contained batch normalization layers, activation layers with leaky Rectified Linear Unit (ReLU) function, and a binary classification layer that utilized cross entropy as the loss function. The detailed architectures of the models are listed in **Table S3**. To simplify the tests, we performed the comparisons for two base models (i.e., CGH model and NLST model). **Figure S5** shows that even though the CGH models developed based on three network architectures are compatible, the NLST models developed based on 2C U-Net significantly outperform those developed based on ResNet-18 and U-Net. These results suggest that the 2C-U-Net has better capability to address anisotropic voxel size and is suitable for clinical chest images. Accordingly, we employed 2C-U-Net for subsequent analyses of population variation and the efficacy of TL.

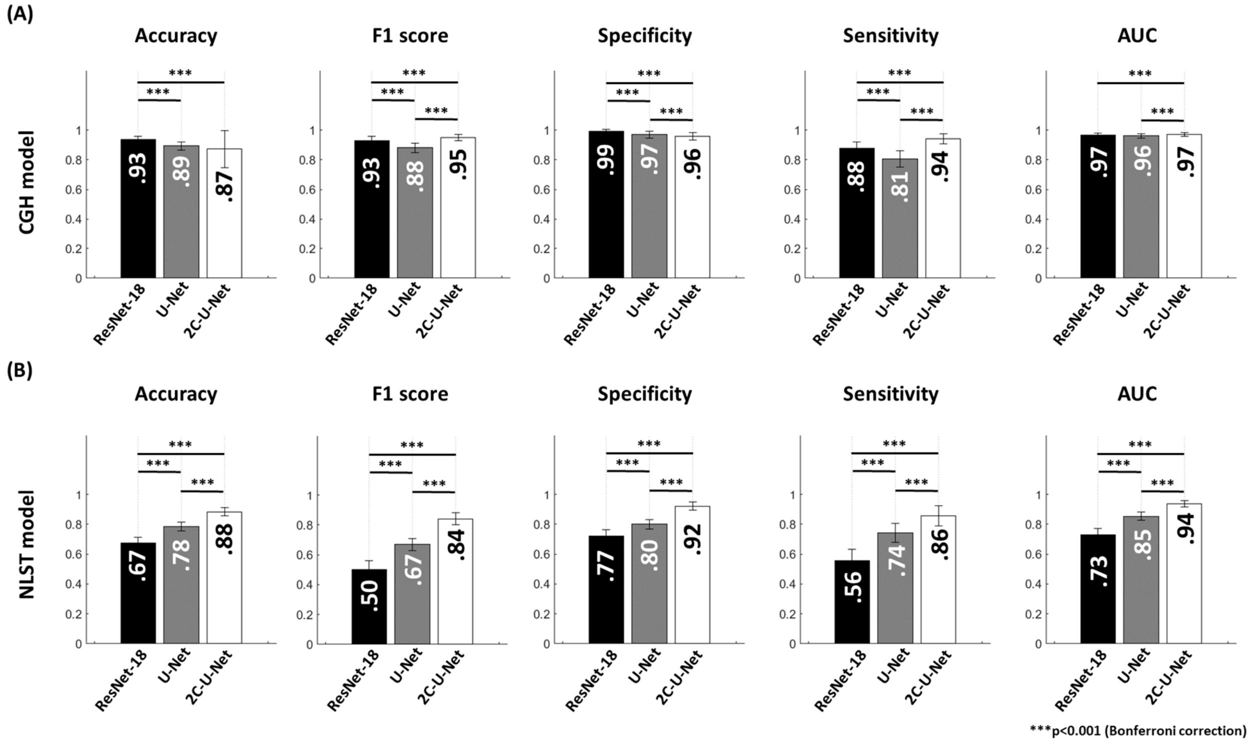

Figure S5. Performance comparisons in the target dataset among ResNet-18, U-Net, and 2C-U-Net. The accuracy, F1 score, specificity, sensitivity, and AUC of the models based on the (A) CGH and (B) NLST test sets, respectively. *** p<0.001 with Bonferroni correction

References:

1. Lee, W.K.; Wu, C.C.; Lee, C.C.; Lu, C.F.; Yang, H.C.; Huang, T.H.; Lin, C.Y.; Chung, W.Y.; Wang, P.S.; Wu, H.M., et al. Combining analysis of multi-parametric MR images into a convolutional neural network: Precise target delineation for vestibular schwannoma treatment planning. *Artif Intell Med* **2020**, *107*, 101911,

doi:10.1016/j.artmed.2020.101911.

2. Ebrahimi, A.; Luo, S.; Chiong, R. Introducing Transfer Learning to 3D ResNet-18 for Alzheimer's Disease Detection on MRI Images. In Proceedings of 2020 35th International Conference on Image and Vision Computing New Zealand (IVCNZ), 25-27 Nov. 2020; pp. 1-6.
3. Çiçek, Ö.; Abdulkadir, A.; Lienkamp, S.S.; Brox, T.; Ronneberger, O. 3D U-Net: Learning Dense Volumetric Segmentation from Sparse Annotation. In Proceedings of Medical Image Computing and Computer-Assisted Intervention – MICCAI 2016, Cham, 2016//; pp. 424-432.
